# Supplementary material for: Perception of healthcare administrators on the impediments of optimizing adverse events following immunization e-Reporting in Nigeria
Source: PLoS One. 2025 Aug 28;20(8):e0331093. doi: 10.1371/journal.pone.0331093 (PMC12393695; doi:10.1371/journal.pone.0331093)
Supplement: Attachment — Submitted filename: Response to Reviewers.pdf [file pone.0331093.s003.pdf]

Response to Reviewers

We appreciate the editorial team and the reviewers for their efforts to help us improve our manuscript to ensure an easy and improved read for the public. We have inserted a table below showing the observations and our responses accordingly

S/N	Comment	Response
**Reviewer 3**		
1	This is an interesting qualitative study exploring barriers to AEFI reporting in Nigeria. The authors write that AEFIs are under-reported in Nigeria (though do not provide supporting evidence). To improve reporting, along with supporting vaccine safety surveillance and properly informing policy decisions, it is important to understand the reasons why AEFIs are not being reported, which may or may not be unique to this country.	Response: Thank you for your observation. This has been addressed. Please see lines 65-68
2	The authors identify four themes/potential challenges to AEFI reporting, though their only recommendation is health worker training.	Thank you for your observation. This has been addressed. Please see lines 519 - 528
3	While overall, this manuscript is generally clearly written, there is some fine-tuning to do and details to iron out. For the Results section, the authors need to stick to the "facts" (strictly reporting) and in the Discussion, the authors might look at other systems that work emphasizing (what can be learned from other countries?), mention anything that is working well (has e-reporting improved AEFI reporting at all?), and really emphasize why this work is important.	Thank you for your feedback. However, after consulting several literature, we found that all extant literature from our search focuses on the challenges that exist in their respective countries and has also made similar recommendations, like in our study. We were unable to identify literature on best practices for AEFI reporting
4	Check references: there are a number that need to be double checked for appropriateness/relevance. For example, references 2-4 (lines 40-45) do not appear to support the claims made in this paragraph. I was unable to see anything about preventing 2-3 million fatalities in reference 3. Reference 4 also does not seem very appropriate, and I am confident the authors can find a study that better supports the sentence. Additionally, references 1,3,14 are all the same – this needs to be corrected.	Response: Thank you for your observation. This has been addressed. The references have been corrected. We also realised that the reference number 4 was meant for another statement in our initial draft, and the referenced statement herein was our own coined statement and not from the literature.
5	Lines 65-72 include information on paper-based reporting. What is the process for e-	Response: Thank you for your observation. Before our study (baseline assessment in

	reporting? Do the health workers report electronically through DHIS-2 and patients self-report through Med Safety? Or can health workers report through either app?	this case), the situation of reporting was such that health workers had only been trained to report electronically through DHIS2, while Med Safety was often advertised to the general public as a reporting tool, because it was initially designed for Adverse Drug Reaction, and was only optimized for COVID-19 to triangulate reporting and also encourage self reporting. The program implementation after our baseline ensured optimal training for both DHIS2 and Med Safety Apps (unfortunately, there is no literature evidence for this scenario). We hope we have been able to clarify this
6	In the Background section, you mention that AEFI reporting is low, below WHO reporting standards (for infants). It would be useful to provide statistics on this. How many AEFIs are reported annually in Nigeria? What is the reporting rate per population? Number or proportion of AEFIs reported electronically and number/proportion reported through the paper form would also be important to include here and gives readers a sense of the "problem". Have rates increased/decreased/remained stable, including after implementing e-reporting? Additionally, are you interested in population-wide AEFIs, or only those associated with infant vaccines? If general population, the infant reporting standard is maybe less relevant.	Thank you for your observation. This has been addressed based on the literature. We would like to add that, there are no accurate figure that has been established in the literature, and even studies from expert such as Omoleke and colleagues (that we have seen), did not provide the identified breakdown (though we would appreciate pointers). This may however be due to the fact that the electronic process is still in its early stages, especially at the time of this study (baseline assessment).
7	Lines 151-158: This paragraph gives some information on participants. It would be helpful to indicate which workers/positions are responsible for AEFI reporting and, if possible, what they do. Is anyone directly involved in or responsible for reporting? I think it would be useful to briefly outline these participants' roles in AEFI reporting.	Thank you for your observation. This has been addressed. Please see lines 172 - 78
8	Lines 161-162: "Two states were chosen from each geo-political zone based on the highest reporting and lowest reporting indicators" – do highest and lowest reporting indicators refer to highest and lowest AEFI reporting? If not, what does this mean?	Thank you for your observation. This has been addressed. Please see lines 180 -84

9	Under "Data Collection", can you include some examples of the questions asked or include a copy of the semi-structured interview questions in an appendix?	Thank you for your observation. Two of the questions asked were already included in the earlier draft, but two more have been added. Please see lines 219 - 23
10	Line 191: "…electronic pharmacovigilance systems defined by Agusoro et al (2018)." I think Agusoro may actually be Agoro et al [17]? Also, how was this paper/study used to make a codebook? Or what was used from the study? Was it the barriers these authors identified?	Thank you for your observation. We have corrected the spelling error. Our study adopted his framework for structuring barriers to pharmacovigilance because we could not identify any relevant framework that could help us scientifically conduct our analysis. Therefore, we borrowed from extant literature.
11	Under "Results", would it be feasible to number participants so the reader knows who is being quoted how many times? (The same person's quotes could be used multiple times throughout the manuscript.)	Thank you for your observation. Our study did not employ the FGD approach, and as such, we could not number the participants. Additionally, we used a generic approach because we want to protect the identity of the study participants as promised, as the team and the participants consider the discussion sensitive to the position of the participants.
12	Under "Results", are any of the participants responsible for reporting AEFIs? The quotes often use 'they', 'those' or 'health workers' instead of first person. It's unclear if anyone interviewed has firsthand, direct experience and can speak to challenges directly. Otherwise, when I read the participant quotes, some of them sound as if they are passing on the blame to other people. It would be important to include the perspectives of those actually doing the reporting.	Thank you for your concern. The scope of our study is to understand the challenges affecting AEFI reporting from a third-party lens (healthcare administrators). This can be observed from our manuscript's topic titled: ***Perception of Healthcare Administrators on the Impediments of Optimizing Adverse Events Following Immunization E-Reporting in Nigeria.***
13	Results for each of the four themes can be pared back a little bit – in some cases it seems like the reporting goes beyond the actual facts/information collected and may be expanding a little too much.	Thank you for your observation. This has been addressed
14	The Discussion does not include a strengths and weaknesses section, which is an important omission.	Thank you for your observation. This has been addressed. Please see lines 479 - 485
15	Lines 412-414: "Additionally, the study found that having the new platform report only AEFIs for COVID-19 was a form of deterrence to health workers, which was also considered a limitation of the reporting system." This is confusing because it sounded like the reporting apps are not actually limited to just COVID and that this was a misconception related to	Thank you for your observation. However, we are uncertain about how our sentence may be confusing to readers. Prior to COVID-19 vaccination, health workers mainly reported childhood vaccination AEFIS. The development and administration of the COVID-19 vaccination led to the documentation of AEFIs for COVID-19 vaccination. As such, health

	lack of training/poor training. This should be clarified.	workers hoped to have the electronic platform provide an opportunity to report both the childhood immunization AEFIs and the COVID-19 vaccination AEFIs
16	Can you comment on the need to go from facility to facility for AEFI reporting? Would it be feasible to report AEFIs without having to visit each site? Is there anyway to streamline the process?	We are uncertain of what you are pointing out here. Kindly elaborate on this.
17	One thing I would like to see in the Discussion is comparison of Nigeria's AEFI reporting to other AEFI reporting systems. Are other countries doing the same thing? Who has a better system?	Thank you for your observation. This is similar to a comment from another reviewer. We found from the literature that several studies with similar settings, like Nigeria, have only been reporting the challenges affecting the AEFI surveillance system in the selected countries. We could not identify literature revealing better AEFI surveillance practices. In line with this, we welcome pointers on how we can get this
18	Recommendation - I agree with this recommendation for continued/ongoing training. However, it sounds like the training hasn't really improved reporting, or has it? Is this the only recommendation? Why not address the other 3 themes/barriers? Is there nothing else that can be improved or changed? If not, it would be good to mention that and why. What other steps or changes can help improve reporting? What parts of the system are working well? .	Thank you for your observation. This has been addressed. Please see lines 519 - 28
19	In the abstract, where it says "…but is probably not causally related to the vaccine" (line 15), I recommend changing this language. Instead of "probably not causally related" I would say something more along the lines of "may or may not be causally related" or even just "may not be causally…".	Thank you for your observation. This has been addressed. Please see line 15
20	In the abstract, under Objective (lines 19-20), I may include that you were identifying barriers to reporting, as that seems to be the focus of this manuscript. If changed, I would include this in the manuscript body as well	Thank you for your observation. This has been addressed. Please see lines 19 - 20 (for abstract), and Lines 100 – 101 (for study background)
21	Also in the abstract, under Conclusion (lines 31-34), I would work on strengthening the conclusion because it currently reads somewhat vague.	Thank you for your observation. This has been addressed. Please see lines 32 - 37

22	Line 62: spell out District Health Information Software 2 the first time and make sure Med Safety App has the proper spacing and capitalization	Thank you for your observation. This has been addressed. Please see lines 79 - 80
23	Study Settings – While this information is interesting, it may not be necessary especially since none of it is used later in the manuscript - I could see it being included if there was discussion about how these factors or differences impact reporting rates or reporting culture, or something along those lines/if later sections build off this information. This section could be shortened considerably.	Thank you for your observation. We do agree with you that the information is not necessary or relevant for this manuscript. However, we consider it to be useful in spreading awareness about the country and the states specifically mentioned in the manuscript. Hence, we have short paragraphs on each state.
24	Line 99: Would be helpful to name the 12 states here in the text.	Thank you for your observation. This already exists in the document. Please see Table 1 (line 161)
25	Under Data Analysis, "The recorded files were equally transcribed verbatim in English language" (lines 185-186), what does "equally" mean here? Additionally, how were the interviews transcribed? Using AI software, a human, etc?	Thank you for your observation. This has been addressed. Please see line 232
26	Line 203: "…approval number NHREC/01/01/2007-19/08/2022" – is this study part of another study? When I tried to look up the NHREC approval number to find out more information, the only thing I can find is that "Patient-reported outcomes of adverse events after COVID-19 vaccination in Nigeria: A mixed methods study" published in 2024 has the same approval number.	Thank you for your observation. However, this error may be coming from the NHREC portal because we have obtained our ethical approval (in 2022) before the development of e-portal for NHREC ethical approval. We have shared the same ethical approval number that was provided to us.
27	Line 216: Table 3 – this table does not seem to add much to the manuscript or reader understanding so I suggest either reporting these numbers in the text or trying to combine with Table 2.	Thank you for your observation. The table has been removed
28	Health workers vs healthcare workers – I would choose one term and try to stick with it consistently throughout the manuscript. Also encourage you to pay attention to capitalization throughout.	Thank you for your observation. This has been addressed
29	Line 261: what exactly is meant by "sensitization program"?	Thank you for your observation. We have added "informative and educative" to denote the meaning of sensitization. Please see line 453
30	Lines 294-296: "…healthcare workers are willing to report and share the AEFI event that has been presented to them in their	Thank you for your observation. However, this interpretation stems from the fact that some of the participants recognized and

	respective facilities but are usually confronted with the challenge of lacking data to synchronize and send out the reported data.” Here, I would remove the bit about workers being willing to report if only they had data plans provided to them. Unless participants actually stated this, we don’t know whether they would be reporting if they had a mobile phone/data, especially given the other barriers to reporting, so this is along the lines of speculation.	highlighted that AEFIs would have been adequately reported electronically but are impeded by a shortage of data (in areas with good network coverage). Kindly refer to lines 309 - 317
31	Lines 296-300: “In other words, the provision of mobile network data to healthcare workers for reporting AEFI electronically will not only serve as a tool for executing their responsibilities, but also as an agent of extrinsic motivation to the healthcare workers, as such mobile data could help them reach out to their co-workers, especially the DSNOs, via social media pages such as WhatsApp.:” I imagine the government/ministry does not want workers to use their work phones or work mobile plans to go on social media. If this would help them reach out to coworkers using a messenger app (such as WhatsApp) for work purposes, I think that would be a different story.	Thank you for your observation. For context, several programme implementations, state and LGA level actors have frequently adopted WhatsApp as a platform for communication and engagement (and in a few cases to discuss data reviews). However, based on your comments, we have added some words to provide clarity “....especially when such platforms are adopted for official purposes, including but not limited to check-in meetings, and communication and social mobilisation”. Please see lines 472 - 73
32	Lines 363-364: “Participants enumerated that the general lack of knowledge of reporting AEFIs in health facilities was driven by negligence on the part of the healthcare workers…” My recommendation here would be tone down this language from ‘negligence’ to something else. This word sounds a little harsh and as though you are placing the blame on the workers when there are other factors at play as well.	Thank you for your observation and feedback. We have refined “negligence” to now read “limited interest”
33	Lines 367-369: “This corroborates the work of Omoleke et al [19] who found the existence of varied and suboptimal knowledge levels of healthcare providers on AEFI definitions and classifications.” The previous sentence talks about japa and it’s unclear how this sentence relates to japa. You need to connect these two ideas better.	Thank you for your observation. Based on your recommendation, we have added a prefix phrase to highlight what our sentence means. It now reads as “***Our findings on inadequate knowledge about reporting*** corroborate the work of Omoleke *et al* [15], who found .....”. Please see lines 359 - 60

34	Lines 430-431: "…and tend to over-rely on the government , with the notion that the government through the appropriate health authority is expected help them address their needs." It strikes me as odd to say – who should they be looking to?	Thank you for your observation. We observed that the misunderstanding comes from how we have phrased the sentence (especially because it is very lengthy). It has now been rewritten to read "Therefore, reneging on promises made to healthcare workers is a derogatory demeanour from the government toward healthcare workers who possess high hopes in seeking redress to their problems, and putting their trust in the government's promises to help them address their needs, through the appropriate health authority.". Please see lines 423 - 428
