# Supplementary material for: Perception of healthcare administrators on the impediments of optimizing adverse events following immunization e-Reporting in Nigeria
Source: PLoS One. 2025 Aug 28;20(8):e0331093. doi: 10.1371/journal.pone.0331093 (PMC12393695; doi:10.1371/journal.pone.0331093)
Supplement: Attachment — Submitted filename: Response to Reviewers.docx [file pone.0331093.s002.docx]

**Response to Reviewers**

Dear Editor and Reviews

Thank you for taking the time to review our paper titled ***Perception of Healthcare Administrators on the Impediments of Optimizing Adverse Events Following Immunization E-Reporting in Nigeria*.** We sincerely appreciate your thoroughness in reading our manuscript and detailed breakdown of feedback to improve our manuscript into a well-accepted article and audience-friendly readable text.

We have taken into consideration all the feedback provided to us in the review outcome and incorporated all that feedback accordingly. Additionally, for some of your comments, we have also explained why we did things the way they are in the manuscript. Below are your comments and our responses (in bold text) to each comment from the reviewers

**Editor’s Comment**

We note that Figure 1 in your submission contain [map/satellite] images which may be copyrighted. All PLOS content is published under the Creative Commons Attribution License (CC BY 4.0), which means that the manuscript, images, and Supporting Information files will be freely available online, and any third party is permitted to access, download, copy, distribute, and use these materials in any way, even commercially, with proper attribution. For these reasons, we cannot publish previously copyrighted maps or satellite images created using proprietary data, such as Google software (Google Maps, Street View, and Earth). For more information, see our copyright guidelines: <http://journals.plos.org/plosone/s/licenses-and-copyright>.

**Response: Thank you for your observation. However, this was produced by our team (within our organization) and was not copyrighted in any way.**

**Reviewer #1**

Clarity and Structure: The manuscript would benefit from clearer organization, particularly in the results section. Subheadings for each theme are helpful, but consider summarizing key findings at the beginning of each section to guide readers.

**Response: Thank you for your observation. This has been addressed**

Thematic Analysis: While the thematic categories are well-defined, the transitions between themes could be smoother. Consider providing a brief summary of how each theme relates to the overall findings, highlighting interconnections.

**Response: Thank you for your observation. This has been addressed**

Literature Integration: Integrate more references to existing literature on AEFI reporting challenges, within similar contexts. This would strengthen the manuscript by situating your findings within the broader research landscape.

**Thank you for your observation and feedback. However, the major challenge is that there are limited papers on the challenges of electronic reporting of AEFis, especially for COVID-19 at the time of writing this paper.**

Technical Details: Provide more detail on the technological barriers mentioned. For example, discuss specific mobile technologies or platforms that could be improved or better utilized to enhance reporting.

**Response: Thank you. This has been addressed**

Participant Diversity: Consider discussing the diversity of respondents in more detail. Addressing the range of perspectives from different regions, roles, or levels of experience could enrich the analysis.

**Response: Thank you very much for your observation and feedback. We do see value in your recommendation. However, the challenge is that the participants socio-demographics were not collected. Hence, it would be difficult to consider such interpretational analysis. Hence, we would consider this in our other papers going forward (depending on the applicability)**

Cultural Context: Incorporate a discussion of the cultural factors that may influence healthcare workers’ attitudes toward reporting AEFIs. Understanding local contexts can provide deeper insights into the identified barriers.

**Response: Thank you for your feedback. This is appreciated. Nevertheless, we would like to remind the reviewer that, this assessment was not directly with the health workers but the health administrators who shared their perception of reporting AEFIs based on the experiences shared with them by the health workers and in some cases, their own experience. Hence, there is little to what we can interpret in this context. This is particularly because we did not directly assess individuals’ self reporting or health workers reporting documentation experiences.**

Training Programs: Elaborate on existing training programs related to AEFI reporting. Discuss their effectiveness and any gaps that exist, which can inform recommendations for future training initiatives.

**Response: Thank you for your feedback. To the best of our knowledge, there are no structured or institutionalized training programs for health workers on AEFI reporting. We have merely made this recommendation because we believe it will be helpful. Training for health workers in Nigeria is often based on program implementations, but the current reality of improved vaccination for disease prevention and cure increases the chances of AEFI reporting, hence why we made our recommendation through our understanding of the study implication**

Government Role: Provide a more nuanced discussion of the government's role in supporting AEFI reporting. Explore how policy changes or increased funding could facilitate improvements in reporting systems.

**Response: Thank you for your feedback. This has been addressed in a separate section titled “Implications of study findings”**

Follow-Up Mechanisms: Address the need for follow-up mechanisms after reporting AEFIs. Discussing how feedback loops could enhance the reporting process might encourage more healthcare workers to engage in the system.

**Response: Thank you for your feedback. This however was addressed in the program implementation that followed our landscape assessment. We would like to remind the reviewer that this manuscript is only a fraction of a whole program implementation, generated from the assessment carried out to understand the peculiarities of AEFI reporting in Nigeria before the actual program implementation**

Technology Adoption: Discuss barriers to technology adoption among healthcare workers in more depth. Exploring attitudes towards technology and previous experiences with reporting systems could provide a clearer picture of the challenges.

**Response: Thank you for your feedback. The scope of our study did not cover barriers to technology adoption or attitudes towards technology, we only assessed health administrators’ perception of challenges associated with electronic reporting of AEFI among grassroots health workers.**

Visual Aids: Consider including visual aids, such as charts or diagrams, to illustrate key themes and findings. Visual representation can enhance understanding and engagement with the material.

**Response: Thank you for your feedback. This has been addressed**

Stakeholders’ Engagement: Highlight the importance of stakeholder engagement in the implementation of recommendations. Discuss how involving healthcare workers, policymakers, and community leaders can foster a more collaborative approach to addressing barriers.

**Response: Thank you for your feedback. This has been addressed in the study conclusion.**

Long-Term Strategies: Propose long-term strategies for sustaining improvements in AEFI reporting. This could include establishing a continuous training framework or integrating reporting into regular health system assessments.

**Response: Thank you. This has been addressed**

Case Studies: If applicable, include brief case studies or examples of successful AEFI reporting systems from other regions or countries. This could provide a model for potential improvements in Nigeria.

**Response: Thank you for your feedback. Should the partners be open to it, we will consider this recommendation when writing about the success of our program implementation. However, in the context of this paper, this is not applicable.**

Language and Terminology: Ensure that technical terms and acronyms (like AEFI) are defined upon first use to make the manuscript accessible to a broader audience, including non-specialists.

**Response: Thank you for your feedback. This already exist in the paper**

Ethical Considerations: Include a brief discussion of ethical considerations related to conducting interviews and reporting on sensitive issues in healthcare. This adds credibility to the research process.

**Response: Thank you for your feedback. If we understand you correctly, our recommendation involves further discussion of confidentiality of the topic or the subject matter. We believe a deeper discussion on confidentiality would not be necessary because our research is from a third-party perspective and not directly with people affected by AEFIs**

Call to Action: Conclude with a strong call to action that emphasizes the urgency of addressing the barriers identified. This can help mobilize stakeholders and create momentum for change.

**Response: Thank you for your feedback. This has been addressed in the conclusion**

**Reviewer #2:**

The study appears to have technical soundness in several aspects but require to enhance methodological rigor and data presentation in order to alignment with publication standards. The choice of qualitative approach for exploring barriers to AEFI e-reporting allows for in depth insights for understanding nuances in reporting challenges. Information from stakeholders at both national and sub-national levels provides with a range of perspectives. The thematic analysis approach categories responses into well defined themes which is methodologically sound for qualitative studies. The sample size of 32 participants is reasonable for qualitative study. But, manuscript lack detailed descriptions of sampling method, interview protocols and specific data analysis processes. For instance, more information on participant recruitment criteria, sampling justification and interview guide content would improve the technical soundness of the study. The manuscript could also provide a clearer summary of data to support each theme, perhaps in a table. The discussion of how themes relate to existing literature is limited while incorporating a comparison with other studies strengthens the validity of findings. The conclusions align reasonably with findings, however, more direct references to participants' statements in relation to each conclusion would improve coherence and credibility. Ethical adherence is noted but explicitly discussing the informed consent process and any steps taken to reduced bias like training interviewers or using triangulations enhance transparency.

**Response: Thak you for your feedback. This has been addressed**

This manuscript is based on qualitative research and does not appear to involve quantitative data or statistical analysis. Instead, it employs thematic analysis, which is a standard approach for analyzing qualitative data. o While the manuscript mentions categorizing responses into themes, it would benefit from a more detailed description of the process, such as whether multiple coders were involved, how consistency in coding was ensured, or if any coding reliability measures were used. A clear explanation of coding process and any efforts to ensure consistency and examples of hoe the themes were derived from raw data improve rigor expected in qualitative analysis.

**Response: Thank you for your feedback. Some of the concerns you raised already exist in the paper, but we have also addressed other that were not**

The manuscript is generally presented in an intelligible manner and uses standard English. It communicates the study's purpose, methodology, findings, and conclusions clearly. However, there are areas where the readability and clarity could be enhanced to meet publication standards fully. Adding clarifications for technical terms and more participant quotations would further enhance the intelligibility and engagement for readers. For Example, Some technical terms and concepts, such as “pharmacovigilance” and “thematic analysis,” could benefit from brief clarifications or definitions, particularly where these terms are first introduced.

**Response: Thank you. This has been addressed**

While the data are technically available upon request, making de-identified data more accessible in a repository would strengthen the manuscript's adherence to open-access standards and facilitate reproducibility. If only summaries of the data (rather than full transcripts) can be shared, this should be specified along with instructions on how to request access. If ethical constraints prevent sharing full data, the authors should explain these limitations explicitly in the data availability statement and clarify that the data meet ethical standards regarding privacy and confidentiality

**Response: Thank you for your observation. We share similar sentiments with you concerning making our data more open access. However, we would like to point out that the nature of our study makes this complicated. This manuscript is a sub-set of a landscape assessment conducted to optimize the AEFI surveillance and reporting system in Nigeria through a collaborative partnership among the Federal Ministry of Health, the National Primary Healthcare Development Agency, and Sydani Group, and funded by Bill and Melinda Gates Foundation (BMGF). Before the landscape assessment, an agreement was made with the Federal Ministry of Health, which is also the Institutional Review Board (IRB) custodian for all health-related research activities in Nigeria. The agreement includes a non-disclosure of facility-related information, which are heavily contained in the transcripts from the assessment. This is also why we could only write from a section of the assessment (because we believe it will be relevant to other developing countries). Providing the data as an open access will be considered a threat to national security by the two government health authorities in Nigeria. Hence, we would appreciate the journal permitting us only to provide our transcripts on a case-by-case request basis. Authors who were involved in the manuscript development and the project implementation (data access committee) will facilitate requests to share the data from the national-level stakeholders should the need arise (see contact details below).**

**Additionally, the funding body also highlighted in the project contract that all data obtained from the project are proprietary of the national health coordinating body and should only be utilized by our organization or any other third party upon agreement with the coordinating body.**

**The contact address that can be reached should the data be required is as follows**

**Organization name: Sydani Institute for Research and Innovation**

**Contact Person: Saheed Dipo Isiaka (email:** [**saheed.isiaka@sydani.org**](mailto:saheed.isiaka@sydani.org)**)**

**Contact Person 2: Grace Erokosima (email:** [**grace.erekosima@sydani.org**](mailto:grace.erekosima@sydani.org)**)**

**Contact person 3: Genevieve Ozioko (email:** [**Genevieve.ozioko@sydani.org**](mailto:Genevieve.ozioko@sydani.org)**)**

**Contact person 4: Queeneth Chigozie (email:** [**queenethguzie@gmail.com**](mailto:queenethguzie@gmail.com)**)**

Please respond to the following queries regarding Figure 1:

Where did the authors obtain the maps, basemaps, shapefiles, map data, etc in Figure 1?

**Response: Having discussed with the team, I have been informed that the basemap was obtained from** [**PPTmaps.com**](https://www.pptmaps.com/) **(a website for editable maps)**

What software was used to make these maps?

**Response:** M**icrosoft PowerPoint was used to design (edit appropriately) the map**
